# Schnitzler’s syndrome - a novel hypothesis of a shared pathophysiologic mechanism with Waldenström’s disease

**DOI:** 10.1186/s13023-019-1117-2

**Published:** 2019-06-22

**Authors:** FS van Leersum, J Potjewijd, M van Geel, PM Steijlen, M Vreeburg

**Affiliations:** 1 Department of Dermatology, Maastricht University Medical Centre, P. Debyelaan 25, 6229 HX Maastricht, The Netherlands; 2 Department of Internal Medicine, Division of Clinical and Experimental Immunology, Maastricht University Medical Centre, Maastricht, The Netherlands; 3 Clinical Genetics, Maastricht University Medical Centre, Maastricht, The Netherlands; 4 Grow Research School for Oncology And Developmental Biology, Maastricht University Medical Centre, Maastricht, The Netherlands

**Keywords:** Schnitzler’s syndrome, Waldenströms macroglobulinemia, Autoinflammatory disease, Hypothesis, Interleukin-1, *MYD88*, *NLRP3*

## Abstract

Schnitzler’s syndrome is an auto-inflammatory disorder which is characterized by two mandatory features: an urticarial rash and a monoclonal gammopathy. Although the pathophysiology of this syndrome is not yet fully understood, a role for interleukin-1 seems apparent. While this presumed link between interleukin-1 and the monoclonal gammopathy is not yet elucidated, a mutual factor in pathophysiology however seems likely. Here we present a novel hypothesis of a shared pathophysiologic mechanism between Schitzler’s syndrome and monoclonal gammopathy.

## Introduction

Schnitzler’s syndrome - as first described in 1972 - is a rare disorder which is diagnosed in the presence of two mandatory clinical features: an urticarial rash and a monoclonal IgM gammopathy or, less common, an IgG gammopathy. These mandatory features are accompanied with at least two of the minor criteria which are listed in Table 1 [1].

**Table 1:** Clinical features of Schnitzler’s syndrome

Major Criteria	
• urticarial rash • monoclonal IgM gammopathy (IgG less common)	
Minor Criteria	
• recurrent fever • arthralgia or arthritis • bone pain • lymphadenopathy • hepato- and/or splenomegaly • elevated ESR and/or leucocytosis • bone abnormalities	

*Diagnostic criteria for Schnitzler’s Syndrome: the diagnosis is made when two major criteria are combined with at least two minor criteria*

The exact prevalence of Schnitzler’s syndrome is not known, although it is thought to be an underdiagnosed syndrome [2]. Since 1972 approximately 200 cases can be found in literature. The male/female ratio has been calculated as 1.76 with a mean age of onset at 51.6 years (+/− 10 years) [2]. Approximately 15–20% of patients with Schnitzler’s syndrome will eventually develop a lymphoproliferative disorder like Waldenström’s macroglobulinemia. This percentage is comparable with the expectancy in patients with a monoclonal IgM gammopathy of unknown significance (MGUS) [2, 3]. There is no presumptive evidence available that Schnitzler’s syndrome is a familial disorder (in contrast to known familial clustering of Waldenström’s disease (WD) [4]. Only one personal communication is known in literature in whom a relative of a patient with Schnitzler’s syndrome was known to have a monoclonal IgM gammopathy [5]. Treatment of Schnitzler’s syndrome has been difficult and unsatisfactory in the past, partly because of the unknown pathophysiology of this disease. Since Anakinra - an IL-1 receptor antagonist – proved to be effective in a case of refractory Schnitzler’s syndrome, a dominant role for interleukin-1 (IL-1) seems apparent [6]. To date this putative correlation between IL-1 and the presence of the paraprotein is not yet fully understood. Here, we present a novel hypothesis which might elucidate the correlation of Schnitzler’s syndrome with MGUS and WD, through a possible mutual pathophysiologic origin.

## Background

The origin of the autoinflammatory character of Schnitzler’s syndrome remains poorly understood. IL-1 involvement seems likely because of the positive reaction to anti-IL1 treatment. In the future it might even be possible to treat Schnitzler patients with long-acting IL-1β antagonists in which 4–8 weekly administration could be equally successful compared with daily dosing as is used in case of Anakinra [7].

A dominant role for IL-1 and the efficacy of anti-IL-1 treatment is also seen in other autoinflammatory diseases like e.g. Cryopyrin-Associated Periodic Syndrome (CAPS). In these patients, either a somatic or germline mutation in the *NLRP3* gene can be found resulting in a spontaneous increase of IL-1β activation by cleaving pro-IL-1β into its activated form. A mutation of the *NLRP3* gene in Schnitzler’s syndrome might therefore be suspected. Such a mutation in this gene is however not always present in Schnitzler’s patients. Only a few Schnitzler patients with severe clinical phenotypes have been described with a proven *NLRP3* gene mutation. In these cases the severity of the disease and the different mutations (somatic mosaicisms) seemed to correlate [8]. In the majority of patients, the exact background of the syndrome remains unexplained.

**Fig. 1:**
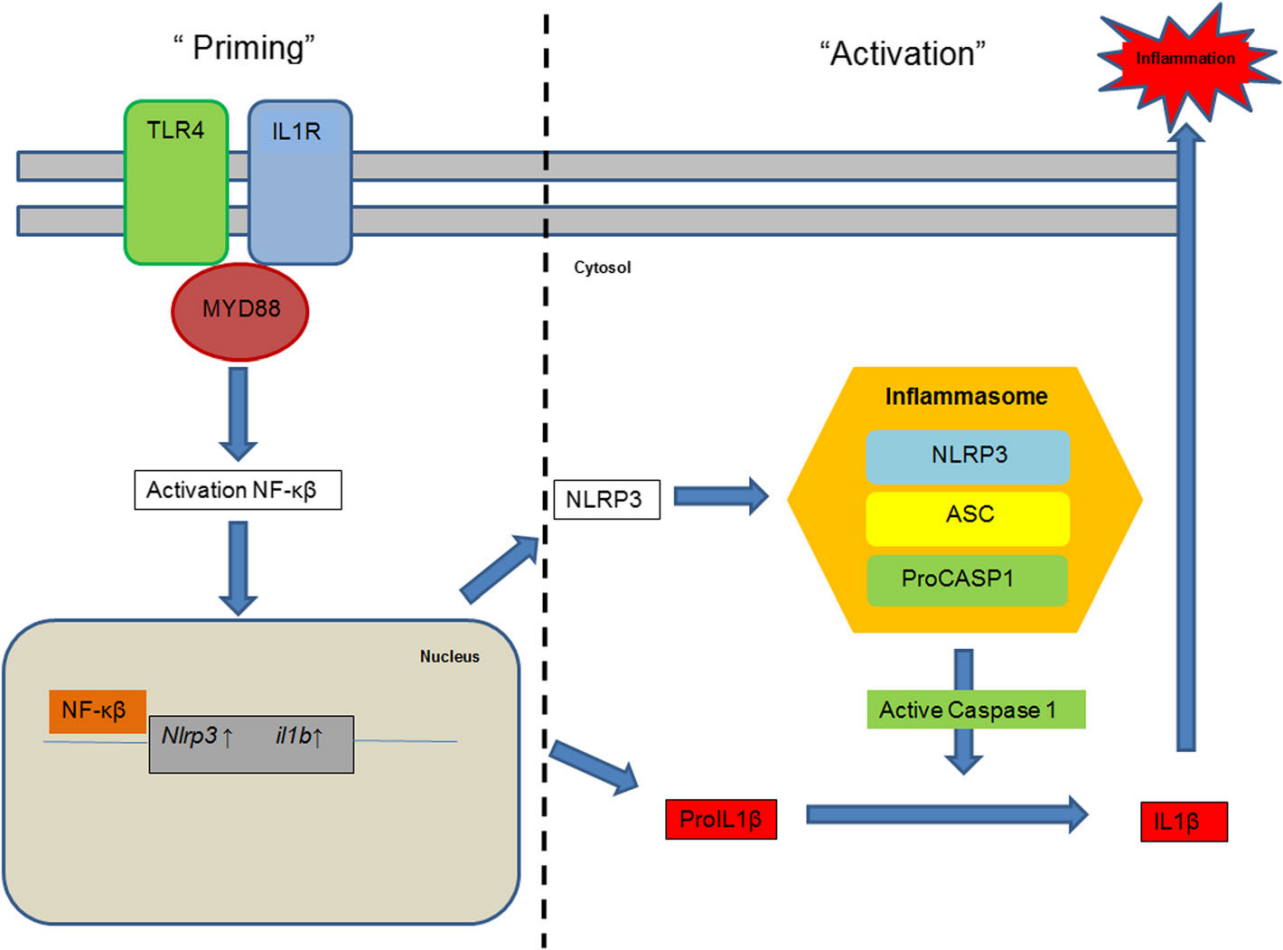
The NLRP3 inflammasome pathway. Here the role of MYD88 as a downstream adaptor molecule in the toll-like receptors and IL-1 receptors is shown in the NLRP3 inflammasome pathway. It has already been proven that MYD88 can cause an induction of NF-κβ which is of importance for the survival of Waldenström’s macroglobulinemia cells. MYD88 serves however hypothetically as a mutual factor in the pathophysiology of MGUS or WD and Schnitzler’s syndrome due to its relation with NF-κβ, NLRP3 and the inflammasome. Furthermore, the increased activity of the inflammasome as seen in Schnitzler’s syndrome might theoretically – via IL1-receptors and MYD88 - increase the dysregulation in the NF-κβ pathway influencing the MGUS or WD

Although there is no direct obvious link between Waldenström’s macroglobulinemia and IL-1 with its associated auto-inflammatory diseases, it still seems likely that MGUS or WD and Schnitzler’s syndrome have a mutual factor in pathophysiology as the latter can not be diagnosed in the absence of a MGUS or WD. Waldenström’s macroglobulinemia is an incurable, IgM-secreting lymphoplasmacytic lymphoma. By performing whole-genome sequencing Tréon et al. [9] described the presence of a specific mutation, p.(Leu265Pro) in the *MYD88* gene in patients with IgM MGUS and Waldenström’s disease. MYD88 is a key downstream adaptor molecule in most Toll-like receptors and IL-1 receptors which can cause an induction of NF-κβ either by ectopic expression [10] or by a gain-of-function mutation in *MYD88,* like p.(Leu265Pro) as described above (see Fig. 1). This NF-κβ signaling is of importance for the growth and survival of Waldenström’s macroglobulinemia cells [9].

Although an alleged Schnitzler’s syndrome without a monoclonal gammopathy has been mentioned before [11], the presence of a monoclonal gammopathy is stated mandatory to accomplish the diagnosis of Schnitzler’s syndrome [1]. In contrast with known Schnitzler’s patients, the MGUS might not be detectable at first consultation. To date the focus on Schnitzler’s syndrome has been on the presence of a *NLRP3* mutation solely, whereas the contribution of MYD88 and NF-κβ signaling has not been intensively investigated yet. Bauernfeind et al. [12] showed that MYD88-mediated signaling can activate the promotor of *NLRP3* and, in case of unique *NLRP3* promotor sequence-variants, can indeed lead to enhanced NLRP3 promotor activity [13]. This dysregulated NLRP3 expression could possibly evoke autoinflammatory symptoms. Increased transcription of both *NLRP3* and *IL-1β* genes due to MYD88 dependent (early phase) NF-κβ activity has been described by Chilton et al. [14]. Furthermore, it was established that MYD88 deficiency and NF-κβ inhibition influence the induction of NLRP3 protein in response to bacterial products (lipopolysaccharides) in a negative manner. This indicates that NLRP3 expression is controlled by signals resulting from NF-κβ activation.

### Hypothesis

Hypothetically, Schnitzler’s syndrome could not be solely a disease primarily caused by a mutation in ‘the inflammasome’-gene (*NLRP3*) but might be a result of the increased NF-κβ activation. This increased NF-κβ activation is also seen in MGUS or Waldenström’s macroglobulinemia. As mentioned before, the presence of a monoclonal IgM is a mandatory criterion for diagnosing Schnitzler’s syndrome. However not every patient with a monoclonal IgM will develop the characteristics of this autoinflammatory disease.

MYD88 can activate the promotor of *NLRP3* and NF-κβ activation seems to control the NLRP3 expression. So theoretically, in case of a MYD88 mutation or increased NF-κβ activation as seen in patients with MGUS or WD - the presence of a certain single nucleotide polymorphism or mosaic mutation in *NLRP3*, may slightly dysregulate NLRP3 which can no longer be compensated. In this hypothesis this will then indeed lead to an increased transcription of pro-IL-1β to activated IL-1β in the inflammasome. Maybe this will eventually result in the clinical presentation of Schnitzler’s syndrome with the presence of monoclonal IgM.

With the abovementioned hypothesis in mind, the increased NF-κβ activation could apparently be controlled to a certain extent lowering the monoclonal gammopathy levels to an undetectable level in some patients. This temporary balance will at some point turn into detectable abnormalities. The influence of NF-κβ activation on the inflammasome may result in elevated levels of IL-1 which could then possibly lead – via IL1-receptors and thus MYD88 – to increasing dysregulation in the NF-κβ pathway (see Fig. 1). By doing so, this will enhance the growth and survival of Waldenström’s macroglobulinemia cells. This however is mere speculation at this time.

## Discussion

The possible link between the presumed role of IL-1 in Schnitzler’s syndrome and the monoclonal gammopathy is to be further examined. It would be of interest to investigate whether and how the treatment with IL-1 antagonists is able to positively influence the presence or progression of the macroglobulinopathy and the associated complications. To date only one patient with Schnitzler’s syndrome, treated with Anakinra showed a reduction of M-protein concentration [15]. In other cases the M-protein levels remained stable during treatment, which lead to the assumption that treatment antagonizing IL-1 has the ability to withhold further growth of plasma cell clones. It is speculated that combining Anakinra with dexamethasone might clear the malignant clone and reduce M-protein levels. This has been illustrated in some patients with indolent malignant myeloma who were at risk for progression to an active myloma [16, 17].

Furthermore, the *NLRP3* gene function is to be assessed in patients with Schnitzler’s syndrome in order to screen for any possible abnormalities or polymorphisms. To our knowledge no *MYD88* analysis has been performed on patients with a known Schnitzler’s syndrome. This analysis, in combination with *NLRP3* analysis in these patients, would be of interest for a better understanding of the pathogenesis of both entities. Besides the abovementioned work-up, a thorough inquiry in patients with WD, concerning the family history for Schnitzler-like manifestations could reveal familial clustering of both diseases. Genetic linkage could be used to investigate the presence of a shared molecular pathogenesis of both entities, however sufficient number of meiosis are essential for this kind of analysis. Haplotype sharing may therefore be a better alternative, but also here, sufficient number of families are necessary for mapping the mutation-containing haplotype. Future research may hopefully lead to a better understanding of the – to this point – enigmatic pathophysiology of Schnitzler’s syndrome.

## Data Availability

Not applicable.

## Abbreviations

CAPS: Cryopyrin-associated periodic syndrome
IL-1: Interleukin-1
MGUS: Monoclonal gammopathy of unknown significance
WD: Waldenström’s disease
